# Can ChatGPT/GPT-4 assist surgeons in confronting patients with Mpox and handling future epidemics?

**DOI:** 10.1097/JS9.0000000000000453

**Published:** 2023-05-10

**Authors:** Yongbin He, Haiyang Wu, Yan Chen, Dewei Wang, Weiming Tang, M. Anthony Moody, Guoxin Ni, Shuqin Gu

**Affiliations:** a School of Sport Medicine and Rehabilitation, Beijing Sport University, Beijing; b Department of Orthopedics, The Fifth Affiliated Hospital of Zunyi Medical University, Zhuhai; c Department of Spine Surgery, Tianjin Huanhu Hospital, Graduate School of Tianjin Medical University, Tianjin; d University of North Carolina Project-China, Guangzhou; e Department of Rehabilitation Medicine, The First Affiliated Hospital of Xiamen University, Xiamen, China; f Duke Molecular Physiology Institute; g Division of Infectious Diseases, Department of Pediatrics, Duke University School of Medicine; h Duke Human Vaccine Institute, Duke University Medical Center, Durham; i Department of Medicine, University of North Carolina Institute for Global Health and Infectious Diseases, Chapel Hill, NC

Highlights

- This is the first study to summarize Mpox-related skeletal and muscular symptoms and provide relevant guidance suggestions for surgeons.
- Mpox needs to be differentiated from common orthopaedic diseases that are also characterized by low back pain and arthritis, and Mpox-related symptoms need to be differentiated from genitourinary symptoms and neurological presentations.
- Surgeons should be vigilant and take appropriate protections to avoid occupational exposure, and be familiar with the guidelines and procedures to achieve optimal results for the patients.
- With ChatGPT’s assistance, surgeons can confidently and calmly confront patients with Mpox and in future epidemics as well.

*Dear Editor*,

Previously known as Monkeypox, Mpox is a zoonosis caused by Mpox virus (MPXV), a linear double-stranded DNA virus belonging to the Orthopoxvirus (OPV) genus. The first human case of Mpox infection was reported half a century ago but only circulated in central and west Africa for a long time. Unexpectedly, Mpox has been rapidly spreading in non-endemic areas through continuous chains of human-to-human transmission since May 2022, sparking concerns worldwide^1^. On 23 July 2022, WHO declared the current Mpox epidemic as a Public Health Emergency of International Concern. The rapid development of artificial intelligence (AI) has resulted in the incorporation of advanced AI technologies into different aspects of our daily lives. OpenAI’s AI-powered chatbot ChatGPT (Generative Pre-trained Transformer) has become a popular topic worldwide and is often highlighted in international news. A growing body of research suggests that ChatGPT/GPT-4 may have significant potential effects in various clinical fields, including public health, infectious diseases, and surgery^2–4^. This article is timely as we witness a global outbreak and because, until now, many surgeons might have been unaware of Mpox, and they are likely to be exposed to these patients in the clinic now or in the future. In this article, we will mainly talk about “Can ChatGPT/GPT-4 assist surgeons in confronting patients with Mpox?”.

## What surgeons should know

**Figure 1:**
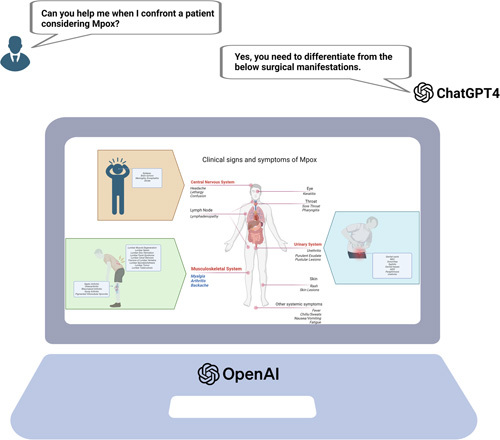
Differential diagnosis Mpox-related surgical manifestations (created with Biorender.com).

Prolonged direct cutaneous contact virion-rich lesion sites is the main transmission mode of Mpox. At present, Mpox transmission predominantly occurs through sexual encounters, with a recent study reporting that 98% of transmission occurred among men who have sex with men. Mpox is a self-limited disease and is classically distinguished into two phases: initial invasive period manifested as fever, headache, cervical lymphadenopathy, myalgia, arthritis, seizure, encephalitis, penile oedema, and so on^5–9^; and a secondary skin eruption phase with typical mucosal and/or skin rash and lesions. However, many cases in the current outbreak have diverse prodromal symptoms and have not presented in a typical staged pattern, especially the arthritis, seizure, encephalitis, and penile oedema caused by Mpox. Mpox-related myalgia (may be low back pain), joint swelling, seizure, encephalitis, and penile oedema may be the first symptoms triaged to surgeons in the outpatient setting. These Mpox-related symptoms need to be differentiated from common orthopaedic symptoms (low back pain and arthritis), genitourinary symptoms and neurological presentations [Fig. 1]. We also have summarized the differences between five common diseases that feature arthritis as a symptom and Mpox-related arthritis in [Table 1]. Surprisingly, when we ask ChatGPT/GPT-4 “If I am a surgeon, what should I know about Mpox?”. The answer from ChatGPT/GPT-4 is relatively comprehensive and organized as shown in [Supplementary figure 1, Supplemental Digital Content 1,http://links.lww.com/JS9/A482]. ChatGPT is a natural language processing model, the more detailed questions you ask, the more detailed the answers you can expect to receive.

**Table 1:** Differences between five most common arthritis and Mpox-related arthritis

Disease	Risk factors	Onset of condition	Clinical presentation	Physical findings	Diagnostic
Septic arthritis	Age >60 years; recent bacteremia; injury or surgical procedure involving the joint; joint prostheses; history of corticosteroid injection; diabetes	Acute or insidious	Hot, swollen, and arthralgia of the knee	Tenderness with limited and severely painful active and passive motion; fever	Laboratory studies including blood tests, blood cultures, joint aspiration with synovial fluid analysis; MRI for detecting irregularities in soft tissue and osseous oedema and identifying coexistent osteomyelitis
Osteoarthritis	Age >65 years; more frequent in women; obesity; trauma; genetics	Insidious	Mild to moderate swollen, pain of the knee	Tenderness and may deformed; reduced range of motion	X-rays may find small spurs called osteophytes at the end of bones; small pieces of cartilage or bone may break off and float inside the joint space
Rheumatoid arthritis	Peak frequency age 20–45 years; more frequent in women; genetics; obesity; heavy smoking	Insidious or acute	Swollen, pain, and stiffness (usually in the morning) of the knee and other joints	Tenderness and may deformed; reduced range of motion; affected multi-joints or on both sides of the body; rheumatoid nodules	Laboratory studies including rheumatoid factor test, joint aspiration with synovial fluid analysis; X-rays may find osteoporosis, osseous erosion
Gouty arthritis	Age >40 years; genetic; more frequent in men; high intake of purines	Insidious or acute	Hot, red, swollen, and agony of the knee	Very tenderness and severely painful active and passive motion; may find tophus	Laboratory studies including uric acid test, joint aspiration with synovial fluid analysis; X-rays may find punch-out lesion at the end of the joint
Pigmented villonodular synovitis	Peak frequency age 30–50 years; more frequent in men; trauma	Insidious	Painless or painful, swollen knee	Tenderness; reduced range of motion	Laboratory studies including joint aspiration with synovial fluid analysis, biopsy; X-rays may find osseous erosion; MRI may find a well-circumscribed or ill-defined soft-tissue mass
Mpox arthritis	Living in or travel through forested areas (specifically near sites habitable to squirrels); sliving in a home with Mpox; high-risk sexual behaviour, especially MSM	Acute	Painless or painful, swollen knee	Tenderness; reduced range of motion; may find fever, rash or lymphadenectasis	Laboratory studies including joint aspiration with synovial fluid analysis, real-time PCR^6,7^

MSM, Men who have sex with men; PCR, polymerase chain reaction.

## How surgeons take action

In addition to low back pain, arthritis, penile oedema, and encephalitis, surgeons will confront patients with Mpox who may need surgery in the emergency department. To ensure appropriate treatment for these patients, meanwhile minimizing occupational exposure, the following summarizes useful measures that can be taken by surgeons and specialist nurses.

First, during consultations in the outpatient setting, inquire meticulously to identify patients with a travel history in Mpox epidemic areas and to identify those who may have male-to-male sex contact history, especially when their manifestations are nonspecific.

Second, if Mpox is suspected, put the patient in a private room and confirm the diagnosis as soon as possible. If a negative-pressure room is available, it can be used. Have the patient avoid contact with other patients.

Third, sterilize hands and wear gloves during the physical examination of patients with suspected Mpox; rash or lymphadenectasis is supportive of a Mpox diagnosis. Personal protective equipment (PPE), including a plastic apron, waterproof surgical gown, and visor, is mandatory. A face filter is also recommended to avoid possible respiratory transmission^10^.

Fourth, an isolation room should be prepared according to the hospital’s infection control guidelines for patients with confirmed Mpox who need to be hospitalized. Inpatient ward safety should be prioritized.

Fifth, for patients who need surgical treatment, elective surgical procedures should be postponed until skin lesions have cleared to avoid increasing the surgical risk. For patients who must undergo emergency surgical procedures or urgent surgical procedures, the following references are based on current international guidelines to ensure the safety of medical workers and other patients. [Supplementary figure 2, Supplemental Digital Content 2,http://links.lww.com/JS9/A483] is the answer from ChatGPT/GPT-4 to the question “If a patient with Mpox needs surgical treatment, how should surgeons take action”. The answer suggests surgeons should consult with a multidisciplinary team, develop a preoperative plan that accounts for isolation precautions, appropriate personal protective equipment, and intraoperative precautions, provide postoperative care, and implement measures for decontamination and waste disposal, surgeons should also be prepared to report cases of Mpox and engage in surveillance activities.

## Perioperative management strategy

In order to reduce the risk of occupational exposure and transmission, the importance of emphasizing perioperative management strategy cannot be overstated. Based on our research and the suggestions from ChatGPT/GPT-4 [Supplementary figure 3, Supplemental Digital Content 3,http://links.lww.com/JS9/A484], we suggest the following:

1. Before surgery: Preoperative discussion should be multidisciplinary and at least include senior surgeons from the trauma department and experts from infectious diseases, anaesthesia, and other relevant departments to fully evaluate the impact of surgery on these patients. Life support is crucial, and nutritional support and aggressive antiviral therapy are necessary.
2. During surgery: A dedicated operating room is ideal for suspected or confirmed cases to reduce dissemination. Level-II PPE is essential for operating room personnel. To reduce operative time and contamination, the operation should be simplified, and the surgery should be completed by a senior surgeon. The surgical procedure must be performed to minimize blood and bodily fluid splashes. After the operation, all the medical personnel must remove and discard the PPE in a designated and marked garbage bin when they want to leave the operating room. Surgical waste must be safely disposed of, and the operating room must be disinfected after the surgery.
3. After surgery: Transfer the patient to an isolation room for specialized treatment as soon as possible after surgery. Complications in patients after surgery can result in serious morbidity and mortality. Some of these complications, including shock or septic shock, deep vein thrombosis, and bronchopneumonia, are more likely during hospitalization, thus, standard-practice preventive measures and treatments should be used.

## Out-of-hospital follow-up suggestions

Patients are suggested to return to the hospital for review 2–4 weeks after discharge for follow-up. A physical examination should be performed to monitor the recovery of the patient’s motor function, and X-rays, computed tomography scans, even MRI can be performed if it is needed. The time of the next follow-up will be determined according to the conclusions from this review. [Supplementary figure 4, Supplemental Digital Content 4,http://links.lww.com/JS9/A485] shows the advice from ChatGPT/GPT4, which is more comprehensive, including wound care; medications; signs of complications; activity and mobility; infection control; follow-up appointments; coordination with public health authorities; and emotional and mental health support.

Mpox spreads rapidly and has become a global public health concern that needs special attention. Luckily, the outbreak seems under control so far. However, even if some countries can eliminate the Mpox virus, other countries such as those in Africa with endemic transmission will remain affected. Although Mpox is rare directly related to surgeons, as a surgeon, it is important to have a basic understanding of the disease to recognize its symptoms and to reduce the risk of occupational exposure and transmission. With the help of the continued developing, powerful ChatGPT, it will be easier and more effective for surgeons to confront the patients with Mpox. What’s more, in addition to Mpox, several infectious diseases such as SARS, H1N1 influenza, Ebola, and COVID-19 have had a significant impact on global health in the past two decades. Unfortunately, there will certainly be other epidemics with significant impacts in the future, and surgeons should be aware of and prepared for such epidemics. Luckily, most epidemics share some common characteristics, and just as surgeons can face patients with Mpox with confidence, we believe that with ChatGPT’s assistance, surgeons can confidently and calmly confront patients in future epidemics as well.

## Ethical approval

This study does not include any individual-level data and thus does not require any ethical approval.

## Source of funding

This work was partially supported by National Natural Science Foundation of China Project (81871848).

## Author contribution

Y.H. and Y.C.: conceptualization, literature search, writing—original draft, collecting the messages, writing—review and editing; H.W.: conceptualization, make the figure and table, writing—original draft; writing—review and editing; D.W.: make the figure and table; W.T. and M.A.M.: review and editing; G.N. and S.G.: conceptualization, writing—review and editing.

## Conflicts of interest disclosure

The authors declare no conflict of interest.

## Research registration unique identifying number (UIN)

1. Name of the registry: Not applicable.
2. Unique Identifying number or registration ID: Not applicable.
3. Hyperlink to your specific registration (must be publicly accessible and will be checked): Not applicable.

## Guarantor

Shuqin Gu.

## Data statement

The data underlying this article will be shared by the corresponding author on reasonable request.

## Acknowledgements

The authors acknowledge that this article was partially generated by ChatGPT (powered by OpenAI’s language model, GPT-4; http://openai.com). The editing was performed completely by the human author.

## Supplementary Material
