# Supplementary figures and images for: Can ChatGPT/GPT-4 assist surgeons in confronting patients with Mpox and handling future epidemics?
Source: Int J Surg. 2023 May 10;109(8):2544–8. doi: 10.1097/JS9.0000000000000453 (PMC10442131; doi:10.1097/JS9.0000000000000453)

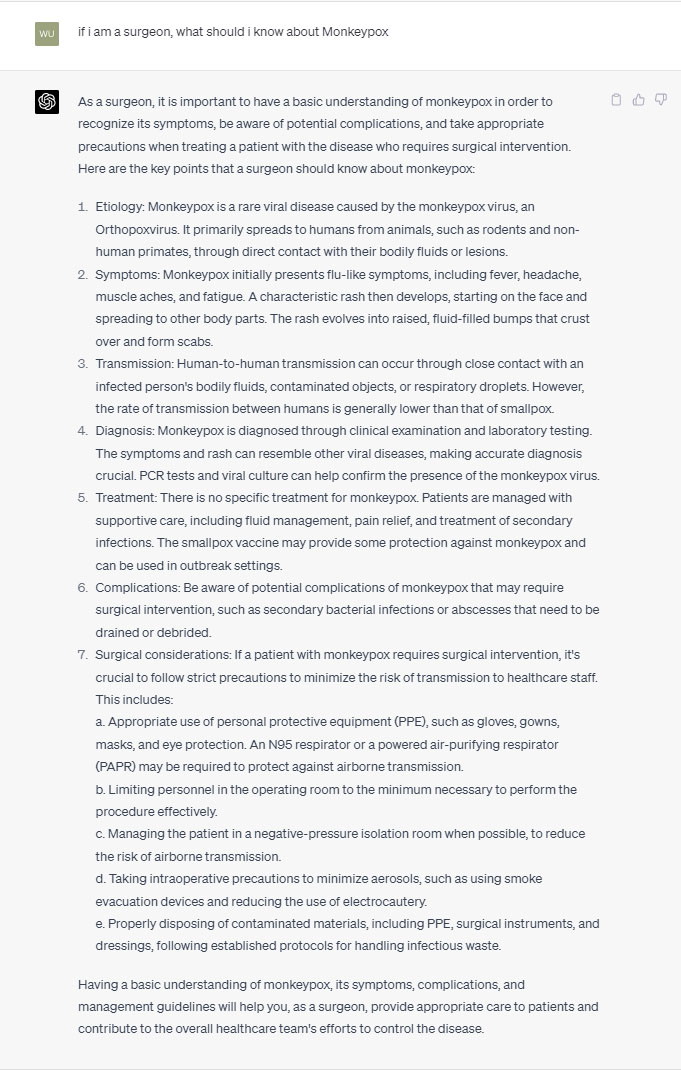

Supplement: Supplementary file 1 [file js9-109-2544-s001.jpg]

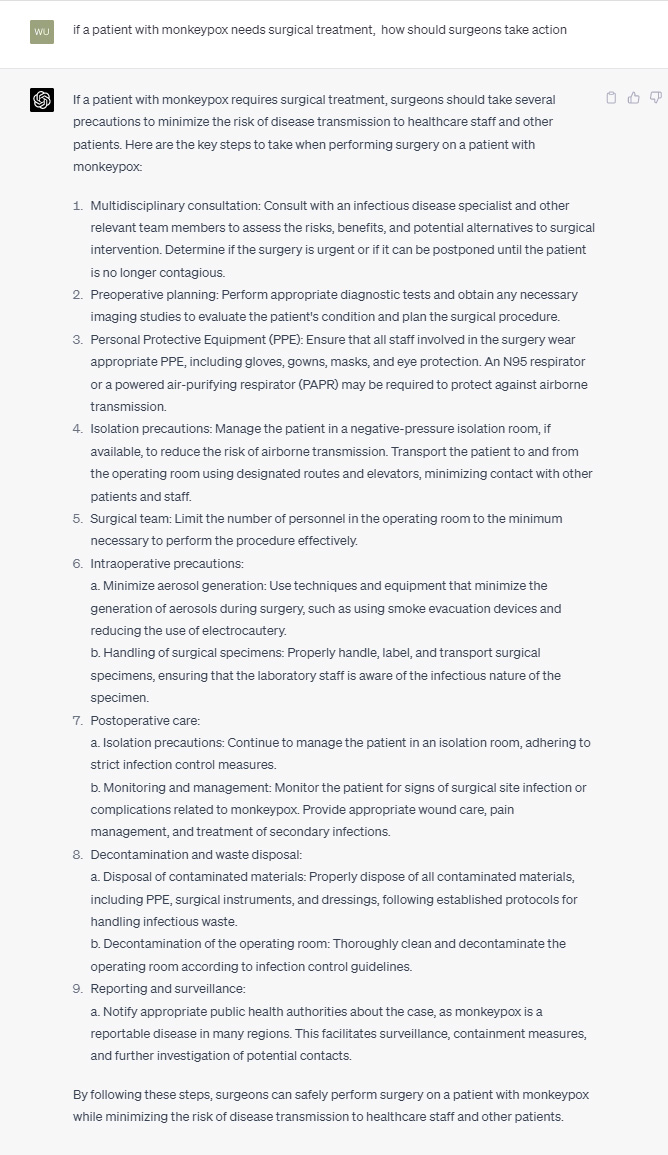

Supplement: Supplementary file 2 [file js9-109-2544-s002.jpg]

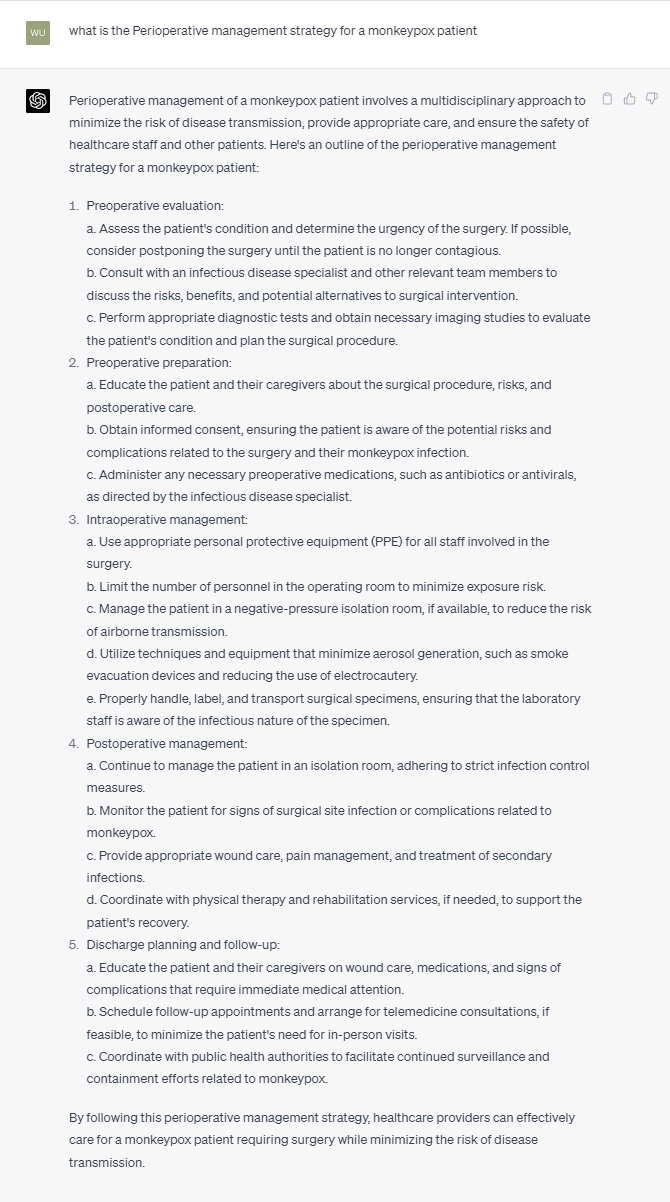

Supplement: Supplementary file 3 [file js9-109-2544-s003.jpg]

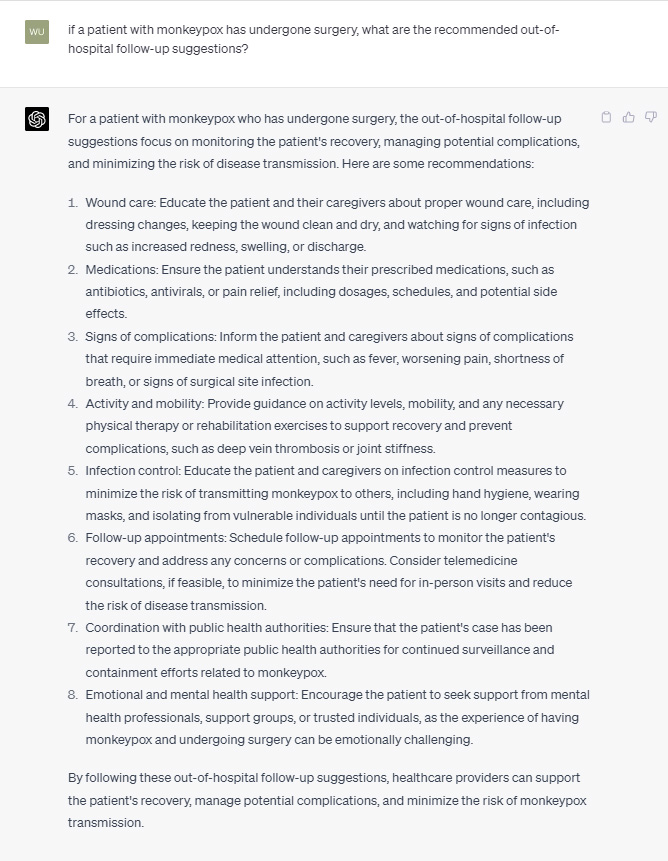

Supplement: Supplementary file 4 [file js9-109-2544-s004.jpg]
